# Long-Term Monitoring of Amphibian Populations of a National Park in Northern Spain Reveals Negative Persisting Effects of *Ranavirus*, but Not *Batrachochytrium dendrobatidis*

**DOI:** 10.3389/fvets.2021.645491

**Published:** 2021-06-21

**Authors:** Jaime Bosch, Amparo Mora-Cabello de Alba, Susana Marquínez, Stephen J. Price, Barbora Thumsová, Jon Bielby

**Affiliations:** ^1^Research Unit of Biodiversity (Consejo Superior de Investigaciones Científicas, Universidad de Oviedo, Principado de Asturias), Oviedo University, Mieres, Spain; ^2^Museo Nacional de Ciencias Naturales-Consejo Superior de Investigaciones Científicas, Madrid, Spain; ^3^Parque Nacional Picos de Europa, Cangas de Onís, Spain; ^4^Genetic Institute, University College London, London, United Kingdom; ^5^School of Natural Sciences and Psychology, Liverpool John Moores University, Liverpool, United Kingdom

**Keywords:** ranaviruses, chytrid fungus, amphibian declines, emerging diseases, population trends

## Abstract

Amphibians are the most highly threatened vertebrates, and emerging pathogens are a serious threat to their conservation. Amphibian chytrid fungi and the viruses of the *Ranavirus* genus are causing disease outbreaks worldwide, including in protected areas such as National Parks. However, we lack information about their effect over amphibian populations in the long-term, and sometimes these mortality episodes are considered as transient events without serious consequences over longer time-spans. Here, we relate the occurrence of both pathogens with the population trends of 24 amphibian populations at 15 sites across a national Park in northern Spain over a 14-year period. Just one out 24 populations presents a positive population trend being free of both pathogens, while seven populations exposed to one or two pathogens experienced strong declines during the study period. The rest of the study populations (16) remain stable, and these tend to be of species that are not susceptible to the pathogen present or are free of pathogens. Our study is consistent with infectious diseases playing an important role in dictating amphibian population trends and emphasizes the need to adopt measures to control these pathogens in nature. We highlight that sites housing species carrying *Ranavirus* seems to have experienced more severe population-level effects compared to those with the amphibian chytrid fungus, and that ranaviruses could be just as, or more important, other more high-profile amphibian emerging pathogens.

## Introduction

Over a quarter of known amphibian species face an elevated risk of extinction (1, 2). Further, many widespread species listed at Least Concern have experienced declines during the last few decades [e.g., (3, 4)]. A growing body of evidence suggests that infectious pathogens have been a major factor in the decline of numerous species and populations (5, 6).

Multiple pathogen types seem to have been involved in these declines, most notably fungi and viruses. Fungi have been highlighted as a major causal agent in vertebrate declines (6–8), with amphibians being severely affected. Viruses are another group of pathogens that have had a disproportionate effect on wildlife health (5), with some species having a high risk of spillover into human populations, with significant impacts on human health (9).

Viruses of the genus *Ranavirus* and fungi of the genus *Batrachochytrium* have had a significant impact on amphibian populations in recent years [e.g., (10, 12)]. The amphibian chytrid fungus, *Batrachochytrium dendrobatidis* (hereafter *Bd*), is a generalist pathogen that has driven declines and extinctions across a broad range of amphibian host species (13, 14). *Bd* is able to infect over 50% of all tested amphibian species, with over 1,000 confirmed host species in at least 86 countries (15). Viral infections caused by ranaviruses (hereafter *Rv*), an emerging group of pathogens with a host range spanning all ectothermic vertebrates, have also become more prevalent and are increasingly associated with mass amphibian die-offs (16, 17). Since *Bd* was described in 1998 (13) the distribution of the fungus has been recorded to include all continents on which amphibians occur (18, 19). Similarly, *Rv* are now found on all continents but Antarctica, although the known geographic distribution remains patchy and, along with the host range of *Rv*, is likely underestimated (20).

The effects of both pathogens vary both inter- and intra-specifically, with impacts ranging from seemingly no effect to population declines (10, 16, 17, 21, 22). Whereas, chytridiomycosis, the disease that may result from *Bd* infection, was quickly recognized as a significant threat to biodiversity, ranavirosis has been slower to gain attention as a threat to amphibian populations. The heterogeneity in individual and population-level response to pathogen exposure means that it is very difficult to ascertain whether the presence of a pathogen, or pathogens, will lead to ill-effects for host-species.

The temporal scale over which disease emergence may affect host populations must be taken into account when attempting to better understand host-pathogen(s) dynamics. Unfortunately, whereas highly visual disease outbreaks in naïve amphibian populations have been recorded, the long-term disease impacts have received less attention, leaving a gap in our knowledge of how infectious disease affects amphibian hosts in the long-term.

The amphibians of Picos de Europa National Park (PNPE) have a long-history of exposure to both *Rv* and *Bd*, and exhibit a great deal of heterogeneity in their response to the pathogens (10). Outbreaks of ranavirosis were first observed in the park in 2005 and resulted in severe and dramatic declines in some species at several locations (10). *Bd* was also found to be present in PNPE shortly after the first mortality incidents associated with ranavirus, although no evidence of chytridiomycosis emergence has been observed (10). Two of the most common species in the park, the common midwife toad (*Alytes obstetricans*) and the alpine newt (*Ichthyosaura alpestris*), can act as drivers of persistence and spread of infection within the community (23). However, although both pathogens are present and life stages of some species harbor infection at high levels, the long-term effects on the host populations remain unclear.

Here, we report the results of long-term monitoring of 24 amphibian populations at 15 sites in a protected area in northern Spain. The monitoring took place for 14 years after an initial disease outbreak of both pathogens (2007–2020). To our knowledge, these analyses represent the first attempt to monitor the long-term effects of *Rv* and *Bd* concurrently in multiple amphibian species and their populations at multiple sites. We used amphibian population data and disease surveillance to identify whether the distribution of pathogens and host population statuses are consistent with long-term disease-related amphibian declines at PNPE.

To do so we aim to answer the following specific questions:

- What are the 14-year trends of the 24 populations in this study?
- Are populations under decline more likely to have experienced mass mortality events, or have *Bd* or *Rv* present compared to populations that are stable?
- Are the prevalence of *Bd* or *Rv* infection higher in sites where mass mortalities were recorded, or in sites containing populations under decline?
- Is the population trend of a population associated with prevalence of pathogen infections?

For these questions we worked with the hypotheses that the prevalence of infection with the pathogens, and the presence of mass mortalities, would be negatively associated with the population status [e.g., (12, 24)].

## Methods

The Picos de Europa National Park (hereafter PNPE) is a protected wilderness area comprised of limestone mountains in the north of Spain. It is an important wildlife area but is also used for recreational activities and stockbreeding. There are nine species of amphibian that can be found in the park, but two of them, the golden-striped salamander (*Chioglossa lusitanica*) and the marbled newt (*Triturus marmoraturs*) occur at only a very small number of locations within the park boundary. This study therefore focused on the remaining seven, more commonly found, species: the fire salamander (*Salamandra salamandra*), the alpine newt (*I. alpestris*), the palmate newt (*Lissotriton helveticus*), the midwife toad (*A. obstetricans*), the spiny toad (*Bufo spinosus*), the Iberian frog (*Rana iberica*) and the common frog (*Rana temporaria*).

### Population Estimates

We comprehensively surveyed 15 sites containing 24 amphibian populations during the amphibian reproductive and development period (May to September) each year from 2007 to 2020 (Table 1). For each species, population monitoring focused on the sites and life-stages that would maximize the probability of obtaining consistently reliable population estimates that would act as a robust measure of population size for that species. For *A. obstetricans* we counted larvae at water bodies instead of terrestrial adults that are difficult to locate. For *S. salamandra* we counted adults crossing roads and/or larvae at water bodies. For alpine and palmate newts (*I. alpestris* and *L. helveticus*) we counted adults at water bodies. For *R. temporaria* we counted egg clutches at small and shallow ponds whereas for *R. iberica* we counted larvae in selected, accessible stream sections. Finally, for *B. spinosus* we counted egg clutches at water bodies and/or adults on roads.

**Table 1:** Results of TRIM models analyzing count data generated in 24 amphibian populations located at 15 sites during a 14 year period.

						**AICc**		**Linear model**			**Additive change**							
**Site**	**Altitude (m), habitat**	**Species**	**Live stage surveillance**	**Number of surveys**	**Serial r**	**Null**	**Linear**	**Slope**	**SE**	**p**	**Parameter**	**SE**	**p**	**Population trend**	***Bd* pos/n**	***Bd*prevalence**	***Rv* pos/n**	***Rv* prevalence**
Bajero	1,865, small lake	*Alytes obstetricans*	Larvae	68	−0.087	31,789.3	13,142.6	−0.0808	0.0245	0.0010	−0.1126	0.0011	<0.0001	Decline	0/69	0.0000–0.0521	8/54	0.0662–0.2712
		*Ichthyosaura alpestris*	Adults	74	0.247	3,198.9	2,732.9	−0.0340	0.0724	0.6391	−0.0704	0.0054	<0.0001	Stable	–	–	–	–
Pozo Llau	1,860, small lake	*Alytes obstetricans*	Larvae	62	−0.126	7,430.1	5,383.5	−0.1815	0.0937	0.0526	−0.1146	0.0044	<0.0001	Decline	2/43	0.0057–0.1581	29/35	0.6635–0.9344
		*Ichthyosaura alpestris*	–	–	–	–	–	–	–	–	–	–	–	–	0/3	0.0000–0.7076	0/3	0.0000–0.7076
		*Rana temporaria*	Egg clutches	50	−0.211	312.98	359.81	0.0150	0.0633	0.8131	−0.0389	0.0148	<0.0001	Stable	2/5	0.0527–0.8534	–	–
		*Salamandra salamandra*	Larvae	59	−0.328	30.1	20.3	−0.1970	0.0804	0.0142	–	–	–	Decline	0/1	–	1/1	–
Lloroza	1,860, small lake	*Alytes obstetricans*	Larvae	72	0.007	11,631.6	3,461.2	−0.3839	0.1081	0.0004	−0.3839	0.1081	<0.0001	Decline	3/48	0.0131–0.1720	21/54	0.2592–0.5312
		*Bufo spinosus*	–	–	–	–	–	–	–	–	–	–	–	–	2/69	0.0035–0.1008	48/76	0.5131–0.7394
		*Ichthyosaura alpestris*	Adults	74	0.371	3795.8	2201.78	−0.1489	0.1099	0.1754	–	–	–	Stable	25/223	0.0739–0.1610	113/226	0.4330–0.5670
		*Rana temporaria*	–	–	–	–	–	–	–	–	–	–	–	–	–	–	0/2	0.0000–0.8419
Andara	1,750, small lake	*Alytes obstetricans*	–	–	–	–	–	–	–	–	–	–	–	–	9/18	0.2602–0.7398	4/12	0.0992–0.6511
		*Ichthyosaura alpestris*	Adults	101	−0.277	119.1	109.25	−0.0521	0.0366	0.1542	−0.0514	0.0157	<0.0001	Stable	–	–	1/1	–
		*Lissotriton helveticus*	–	–	–	–	–	–	–	–	–	–	–	–	–	–	–	–
Moñetas	1,730, small lake	*Alytes obstetricans*	Larvae	69	−0.165	47,797.7	8,975.4	−0.4923	0.0847	<0.0001	−0.4618	0.0103	<0.0001	Decline	0/6	0.0000–0.4593	5/8	0.2449–0.9148
		*Ichthyosaura alpestris*	Adults	67	−0.227	496.6	478.7	−0.0233	0.0292	0.4251	−0.0065	0.0065	<0.0001	Stable	1/22	0.0012–0.2284	5/24	0.0713–0.4215
Cable	1,730, group of ponds	*Ichthyosaura alpestris*	Adults	66	0.153	222.5	946.3	0.0780	0.0949	0.0538	0.0843	0.0085	<0.0001	Stable	0/18	0.0000–0.1853	0/18	0.0000–0.1853
		*Lissotriton helveticus*	Adults	64	0.074	329.2	240.4	0.0869	0.0604	0.1503	0.0225	0.0154	<0.0001	Stable	0/1	–	0/1	–
		*Rana temporaria*	Egg clutches	62	0.001	135.2	133.3	0.0005	0.0253	0.9841	0.0047	0.0068	<0.0001	Stable	0/1	–	0/1	–
Vega Salambre	1,300, pond	*Rana temporaria*	Egg clutches	64	0.019	662.3	931.31	0.0073	0.0413	0.8602	−0.0158	0.0066	<0.0001	Stable	–	–	–	–
Vau los lobos	1,130, cattle tank	*Alytes obstetricans*	–	–	–	–	–	–	–	–	–	–	–	–	0/11	0.0000–0.2849	0/11	0.0000–0.2849
		*Ichthyosaura alpestris*	Adults	39	−0.115	2.7	−3.0	0.0801	0.029	0.0072	0.0775	0.0291	0.2463	Increase	–	–	–	–
		*Lissotriton helveticus*	Adults	38	0.109	113.4	114.0	−0.0106	0.0485	0.8275	−0.0115	0.0124	<0.0001	Stable	–	–	–	–
Rasa Pandecarmen	1,117, pond	*Rana temporaria*	Egg clutches	45	0.425	816.8	3,617.6	0.0985	0.1128	0.3823	–	–	–	Stable	–	–	–	–
Ercina	1,110, lake	*Alytes obstetricans*	–	–	–	–	–	–	–	–	–	–	–	–	72/102	0.6075–0.7920	49/183	0.2051–0.3381
		*Bufo spinosus*	Adults	74	0.104	758.2	79.5	−0.2452	0.0422	<0.0001	−0.2084	0.1096	<0.0001	Decline	2/42	0.0058–0.1616	14/47	0.1734–0.4489
		*Ichthyosaura alpestris*	–	–	–	–	–	–	–	–	–	–	–	–	2/43	0.0057–0.1581	6/43	0.0530–0.2793
		*Lissotriton helveticus*	–	–	–	–	–	–	–	–	–	–	–	–	2/2	0.1581–1.0000	0/2	0.0000–0.8419
		*Rana temporaria*	–	–	–	–	–	–	–	–	–	–	–	–	8/38	0.0955–0.3732	6/39	0.0586–0.3053
La Güelga	1,050, cattle tank and stream pond	*Alytes obstetricans*	–	–	–	–	–	–	–	–	–	–	–	–	33/87	0.2774–0.4897	1/34	0.0000–0.1533
		*Ichthyosaura alpestris*	Adults	42	−0.089	34.8	18.4	−0.173	0.0983	0.0786	–	–	–	Stable	–	–	–	–
		*Lissotriton helveticus*	Adults	63	0.270	824.0	666.0	−0.0723	0.1072	0.4996	−0.0127	0.0093	<0.0001	Stable	–	–	1/1	–
Allende Cabañes	790, stream	*Rana iberica*	Larvae	33	−0.342	556.1	490.6	−0.1995	0.2096	0.3411	–	–	–	Stable	0/2	0.0000–0.8419	0/2	0.0000–0.8419
		*Alytes obstetricans*	–	–	–	–	–	–	–	–	–	–	–	–	0/10	0.0000–0.3085	0/14	0.0000–0.2316
Pontón-Oseja	1,260–750, road	*Salamandra salamandra*	Adults	67	−0.131	426.9	377.4	−0.0558	0.0614	0.3632	−0.0764	0.0137	<0.0001	Stable	0/19	0.0000–0.1765	0/19	0.0000–0.1765
La Guxana	200, stream	*Rana iberica*	Larvae	29	0.068	1,085.9	880.3	−0.1125	0.1520	0.4592	–	–	–	Stable	0/3	0.0000–0.7076	0/7	0.0000–0.4096
		*Bufo spinosus*	–	–	–	–	–	–	–	–	–	–	–	–	–	–	0/13	0.0000–0.2471
Soto Cangas-Covadonga	200, road	*Bufo spinosus*	Adults	40	0.020	2,232.5	209.1	−0.5096	0.0820	<0.0001	–	–	–	Decline	0/20	0.0000–0.1684	17/26	0.4433–0.8279

*Two different models were constructed for each population; a no time-effects models (null; testing for the absence of population trends) and linear trend (assuming an increasing or decreasing monotonic trend). Serial r: serial correlation measuring the association of the counts in year t with year t−1. The AIC figures are provided for each of these models, as is the slope of the linear trend model, its standard error and significance (against Ho: slope = 0). The additive parameter defines the overall additive change in abundance for each species (SE = standard error and the significance of deviation from the null hypothesis of 0 –p–). Population counts remained constant if the additive parameter equaled zero; an additive figure of −0.1126 denotes that from the first to the last year of amphibian counts the population has decreased at an average inter-annual rate of 11.3%. All significant tests remain significant after Bonferroni sequential correction. For each population and pathogen, the number of positive and total samples analyzed, as well the Clopper-Pearson confidence intervals, are shown*.

In order to sample this diverse set of species and different life-history stages at 15 heterogeneous sites a range of survey techniques were required. Surveys included transect walks (*n* = 11), walks along fixed sections of streams (*n* = 2) and car transects along specified sections of roads (*n* = 2). Road surveys for adult amphibians were conducted by a single observer driving at very slow speed at twilight. Water body surveys were completed during the day by a pair of observers traveling on foot at a consistent speed to maintain a standardized sampling effort and counting every animal or clutch. Both observers independently counted every observation resulting in a total estimate of abundance. If one of the counts was more than twice the other, the survey was repeated, otherwise the mean count was used as the abundance. We estimated population abundances up to 12 times per year (mean = 4.7; Table 1). Thus, the methodology and life history stage targeted varied with the characteristics of the study species and the locality but remained consistent for each population within and between years.

A mass mortality incident was defined as an unusually high number (more than five) of dead animals of a single species being recorded at a given site on more than one occasion during a single year.

### Pathogen Diagnostic Sampling

Tail or toe-clips were used for diagnosis of both *Rv* and *Bd*, except for *Bd* in anuran larvae. The latter only become infected in their oral disc, and so for *A. obstetricans* larvae an oral disc swab was taken for *Bd* diagnosis. Since *B. spinosus, R. iberica*, and *R. temporaria* have smaller larvae, oral swabs are not a reliable method of sampling, and tail clips would compromise animal welfare. Individuals were therefore humanely euthanized using an overdose of buffered MS222 (5 g/l), and the whole oral disc was used for detection of *Bd*. All tissue samples were fixed in 70% ethanol in the field. Swabs were kept dry and refrigerated prior to processing in the laboratory.

In terms of sample sizes, for each species individuals were sampled for *Bd*/*Rv* infection exhaustively for those sampled via swabs, and up to a maximum of 20 per population and year in the case of tissue samples of live individuals. Most samples used in this study were collected during the past 5 years. All research was performed in accordance with relevant guidelines and regulations and under license from the PNPE. All experimental protocols were approved by Comisión Ética de Experimentación Animal MNCN-CSIC (reference 666/2018).

DNA was extracted from tissue samples using DNeasy Blood and Tissue Kit (Qiagen, Hilden, Germany) following the manufacturer's protocol. DNA was obtained from swabs with PrepMan Ultra following Hyatt et al. (25). qPCR for *Bd* and *Rv* was performed following Boyle et al. (26) and Leung et al. (27), respectively, on a MyGo Pro PCR machine. Negative controls and standards with known concentrations of *Bd*/*Rv* were used in each plate.

### Statistical Analyses

To obtain population trends through time for further analyses, population monitoring data generated as described above were used in time-effects model. Modeling was performed using TRIM, software developed specifically for time series of animal counts from monitoring programs (28). Log-linear Poisson regression models were used on the species' count data from 2007 to 2020, using the maximum value obtained for each species' population as the best estimate of the population size for that year. The population estimate was included as the continuous predictor and the number or surveys performed each year for a particular population as weights. These models accounted for over-dispersion and serial autocorrelation in the data to obtain population trends (±1 SE) as the slope of the regression of the logarithms of the yearly indices. Standard errors of the trends were estimated as a measure of uncertainty in average linear population trends. We did not use non-linear models as our objective was to investigate overall change during the course of the sampling period and not annual, or other short-term fluctuations in abundance [see also (12, 29) for a similar approach]. Linear trend TRIM models were compared with their corresponding null TRIM models (not including the linear effect of year) using Akaike's Information Criterion (AIC). We estimated the overall additive change for each species by measuring the average inter-annual rate from the first to the last year of the study.

One-tail Fisher's exact tests were used to check whether population trends were associated with observation of mass mortality events, and if the status of amphibian populations were associated with the presence of *Bd, Rv*, or both at the site. To do this contingency tables were constructed using the count of declining and non-declining sites against the count of sites ± mass mortality; ± *Bd* presence; and ± *Rv* presence respectively.

One-tail *t*-tests were used to determine whether there was a significant association between the prevalence of *Bd* and *Rv* and the occurrence of mass mortalities, and also between the pathogen prevalence and the presence of declining populations at a site. To do this, for each site, the prevalence of each pathogen was averaged across all sampled species located in each site. Those sites were then categorized depending on whether one of their constituent species had experienced a mass mortality or not and whether one of their constituent species exhibits a declining population or not.

Finally, we used a general linear mixed model to analyze if the slope of the linear trend models for the 24 studied populations, across species and sites (as random factors), was related to the log-transformed *Bd* and *Rv* obtained values of prevalence. The number of surveys was introduced as a covariate into the analysis, and species was considered as a random factor because different species were surveyed using different life-stages. For populations of species in which the prevalence of *Bd*/*Rv* was not obtained, a value of 0 was assigned if a co-existing population of the higher sensitive species *A. obstetricans* in that site tested free for *Bd*/*Rv*. In other cases the averaged prevalence of *Bd*/*Rv* per species at that particular site was used.

## Results

**Figure 1:**
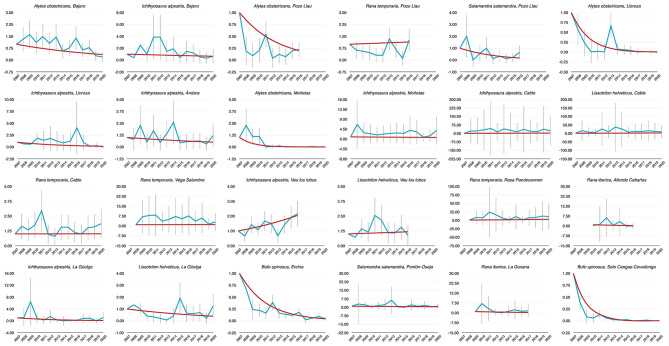
Dynamics of abundance of 24 populations of PNPE estimated from 2007 to 2020. Time index 1 refers to the amphibian counts measured in 2007 (i.e., the baseline in first sampling year). Blue lines are annual abundance estimates, while red lines were established by means of TRIM models.

**Figure 2:**
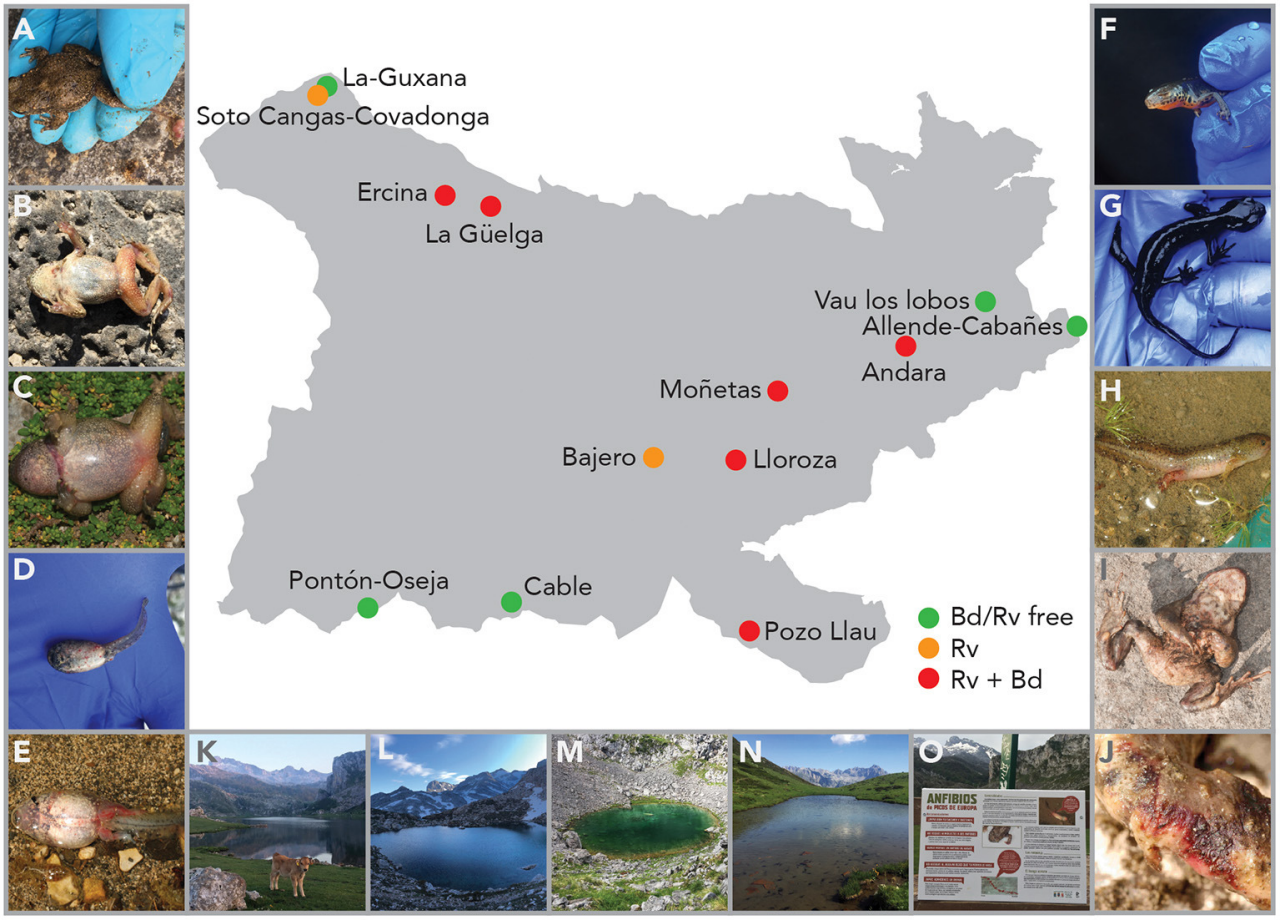
Map of PNPE with sampling sites and signs of ranavirosis in three species. *Alytes obstetricans* adults with severe limb necrosis **(A)** or erythema **(B)**, metamorph with erythema and swelling of the body and legs **(C)**, and larvae with systemic hemorrhage **(D, E)**. *Ichthyosaura alpestris* adults with missing eyes **(F)** or cachexia **(G)** and larvae with systemic hemorrhaging **(H)**. *Bufo spinosus* adults with erythema **(I)** and with open lesion on the feet **(J)**. Study locations of Ercina **(K)**, Lloroza **(L)**, Moñetas **(M)** and Pozo Llau **(N)**, and signage to inform visitors about the presence of *Rv* in PNPE **(O)**.

Population trends over the period 2007–2020 were obtained from TRIM analysis and are outlined in Table 1 and shown in Figure 1. In total data for 24 amphibian populations at 15 sites were collected during this time period, during which 60% of sites held non-declining populations, the remaining 40% of sites held a declining population of at least one species. Declining population trends were obtained for *A. obstetricans* (4/4), *B. spinosus* (2/2), and *S. salamandra* (1/2). Non-declining population trends were obtained for *I. alpestris* (7/7), *L. helveticus* (3/3), *R. iberica* (2/2), and *R. temporaria* (4/4). Mass mortalities were recorded in 47% of the study sites. *Bd* and *Rv* were present in 46 and 62% of sites, respectively. During the time-frame of the study we have consistently recorded mass mortality incidents consistent with ranavirosis in four species: *A. obstetricans, I. alpestris, S. salamandra*, and *B. spinosus* (Figure 2).

There was a significant association between the occurrence of mass mortality and whether or not a site held a population of species in decline: 86% of the sites in which mass mortalities were recorded were home to declining populations (Fisher's exact test, *p* = 0.0014). There were contrasting patterns in relation to whether sites with declining populations had associations with the presence of amphibian pathogens: there was no significant difference in the status of sites with and without declining populations and the presence of *Bd* (declining and *Bd*-positive = 67%; declining and *Bd*-negative = 33%; Fisher's exact test, *p* = 0.2086). On the other hand, none of the *Rv*-free sites were home to declining populations, whereas just 25% of the sites where *Rv* is present are not home to populations which have declined (Fisher's exact test, *p* = 0.0163). These results suggest that sites that have experienced mass mortality are more likely to have declining populations, and that *Rv*, but not *Bd*, is associated with declining populations within a site.

There was no association between the average *Bd* prevalence at a site and the occurrence of a mass mortality in one of its species (0.1542 vs. 0.0632, *t*_11_ = 0.9032, *p* = 0.1929), whereas the prevalence of *Rv* was significantly higher at sites in which a mass mortality was recorded (0.4309 vs. 0.0630, *t*_11_ = 4.5438, *p* = 0.0004). Sites with populations under decline exhibit similar *Bd* prevalence to sites supporting non-declining populations (0.0967 vs. 0.1256, *t*_11_ = 0.2774, *p* = 0.3933), whereas those sites with populations under decline present higher values of averaged *Rv* prevalence (0.4385 vs. 0.063, *t*_11_ = 3.4364, *p* = 0.0028).

The general linear mixed model explained 88% of the variation in the slopes of the 24 studied populations. The slopes are not related to the number of surveys (*F*_1,13_ = 1.9320, *p* = 0.1874) nor the prevalence of *Bd* (*F*_1,16_ = 2.6965, *p* = 0.1199). On the other hand, the prevalence of *Rv* is a key factor in the variation of the population slopes (*F*_1,18_ = 10.5318, *p* = 0.0046): those populations with higher values of *Rv* prevalence present slopes with the largest negative coefficients.

## Discussion

Analyses of 14 years of amphibian population data at multiple sites for multiple species suggest that population declines in this community of amphibians are consistently associated with the presence of ranavirosis. *Bd*, the causal agent of chytridiomycosis, does not seem to be associated with disease emergence or population impacts, which contrasts with the situation in another montane region in Spain, Guadarrama National Park, in which two of nine species exhibited severe population-level effects of chytridiomycosis (12).

It is unclear why *Bd*, which has consistently affected montane communities of similar species in other parts of Europe has not had major population-level impacts at PNPE. One possibility is that *Bd* and *Rv* (which were first detected in 2005) caused declines in the most susceptible species before systematic monitoring of populations began (monitoring started in 2007). The common midwife toad, *A. obstetricans*, is known to be important in the persistence and spread of both *Bd* and *Rv* in amphibian communities (23, 30). If their populations were reduced significantly before 2007, their role in the transmission of *Bd* infection to other species could have been greatly reduced, and hence the impact of *Bd* on sympatric species could have significantly reduced, which is consistent with the lack of strong association between the presence of *Bd* and mass mortalities or population decline. In contrast *Rv*, which can be spread more readily by other species, could continue to drive population reductions even in the absence of *Alytes obstetricans*. Alternatively, and since it has been proved that susceptibility to an emerging pathogen is related to population genetic diversity [e.g., (31)], *A. obstetricans* populations of northern Spain may be more protected than others on the Iberian Peninsula because of their broad distribution and abundance (32).

Three species in the PNPE, *A. obstetricans, B. spinosus*, and *S. salamandra* exhibited declining population trends over the 14 years of the study. All study populations of *A. obstetricans* (4/4) were declining, with some having been practically extirpated. The one population of *S. salamandra* infected with *Bd* and *Rv* has been reduced to very low abundance, whereas the other population—apparently free of infection by either pathogen—exhibited a stable population size over the 14-year period. Both populations of *B. spinosus* experienced sharp declines when infected with *Rv* or with both pathogens. These three species represent the most heavily *Bd*-affected species in Guadarrama National Park (12). *Bufo spinosus* and *S. salamandra*, have been severely affected in PNPE and in Guadarrama NP and appear to be sensitive to both *Bd* and *Rv*. These two species present a broad distribution in the Iberian Peninsula, and have experienced a remarkable decline during the last few decades (33), the drivers of which are poorly understood. However, given their susceptibility to infectious disease it remains a possibility that those declines and extirpations were driven by pathogens.

Four species at PNPE had stable population trends between 2007 and 2020, highlighting the heterogeneity in population-level effects of infectious disease. The palmate newt, *L. helveticus*, and common frog, *R. temporaria*, look to be unaffected by both pathogens at the population-level. Most populations remain free of both pathogens, and when infected, their population trends are stable. This finding in respect of *R. temporaria* is somewhat surprising given that ranavirosis in this species has been observed in this species and the severe impact it has had on the species in England (11, 21). However, common frogs in England are mostly infected by frog virus 3-like ranaviruses (34, 35) whilst common midwife toad ranaviruses circulate in PNPE (10). In addition, the severe effects of ranavirosis in English frogs have generally been observed in populations in residential garden ponds (35, 36), which provide a very different environment to that observed in the protected area of the PNPE. It is therefore unclear whether viral genetics, environmental effects or some other factor explains the different outcome for *R. temporaria* in the PNPE. The two study populations of *R. iberica*, even when they are present in very small numbers, also remain stable and free of both pathogens.

The final species monitored in this study seems to have had a more complex history of dynamics with these pathogens. The alpine newt, *I. alpestris*, which experienced recurrent mass mortalities and sharp declines during the initial period following *Rv* emergence (10), is apparently recovering at sites housing infected individuals. At the population-level, *I. alpestris* has remained stable over 14 years, and populations are increasing in some locations that are free of pathogens. These results are broadly similar to those recorded at Guadarrama National Park (12): even when most larvae and adults are infected with *Bd* (37), the species continues to expand its distribution and population size soon after its introduction in the area (38). Again, an explanation for the apparent rebound of *I. alpestris* during the last years could be the sharp decline of *A. obstetricans*. Adult *I. alpestris* were observed often in the area feeding on moribund and heavily *Rv* infected larvae of *A. obstetricans* during the first years after ranavirosis emerged. These animals soon presented facial hemorrhaging, swollen skin around the mouth, problems opening jaws and even loss of eyes. In recent years, when no moribund *Alytes* tadpoles were present in the water, adult *I. alpestris* exhibited less severe and less frequent signs of ranavirosis (J. Bosch, personal observations) despite *Rv* infection being persistent in the species. Over-wintering larvae are known to be an important host for many infectious pathogens, including *Bd* and *Rv* (23, 39–41), and so perhaps with a reduction in their abundance other species can benefit from more breaks in the chain of initial transmission and subsequent re-infections.

The long-term outlook for amphibian populations in PNPE remains unclear; our results suggest that *Rv* and not *Bd* is more closely associated with population declines in the past 13 years. Our results are similar to those obtained in other, similar communities in the Iberian Peninsula (e.g., the species exhibiting the highest prevalence levels and susceptibility to decline), but vary in others (the importance of the pathogens in question). The effects of both pathogens can be highly context-dependent, with declines perhaps being driven by interactions with other important factors including climate (11, 42); ozone levels (43); and microbiome (44, 45) of hosts; and the specific ecological context in which the host community lives (23). All of these factors are likely to interact with pathogens as a driver of population-level effects and warrant careful attention both alone and in combination with each other.

Altitude appears to be the key factor driving *Bd* infections in Iberia [e.g., (46)] and dictating chytridiomycosis outbreaks (47, 48), but in our study area mass mortalities occurred across a broad range of altitudes (from 200 to 1,865 m). Within this range the accessibility of the sites to human traffic varies greatly, which could affect the likelihood of pathogen introduction and spread: those study sites with low visitor numbers appear to be the ones free of one or both pathogens. PNPE receives more than two millions visitors per year, with a great number of visitors coming from overseas. Unfortunately, no biosecurity measures have been adopted to avoid pathogen or pest introductions. Among other things, encouraging visitors to undertake biosecurity hygiene practices such as cleaning footwear is desirable, and possibility of installing physical barriers at some sites to avoid visitors coming into direct contact with water and moving animals between sites could also be a positive step in preventing the spread of pathogens.

This study highlights the long-term, persistent effects of pathogens of the genus *Ranavirus* on amphibian populations and communities. Over a 14 year period *Rv* was consistently associated with declines, whereas *Bd* appears to have a weaker, if any association. Long-term datasets and their analyses provide a means to identify and highlight host-pathogen dynamics that are not immediately obvious, or may even be missed entirely from shorter-term studies. While *Bd* is still heralded as the major disease threat to amphibians [e.g., (49)], long-term site-level surveillance is often not included in the related research. Our results suggest that, in addition to large-scale comparative analyses, longer-term, more detailed studies encompassing a broader range of pathogens may prove informative for planning conservation action.

## Data Availability Statement

The raw data supporting the conclusions of this article will be made available by the authors, without undue reservation.

## Ethics Statement

The animal study was reviewed and approved by Comité de Ética, CSIC.

## Author Contributions

JBo: conceptualization, funding acquisition, investigation, methodology, data curation, formal analysis, writing - initial draft and review and editing. AM-C and SM: data curation and writing - review and editing. SP: writing - review and editing. BT: formal analysis and writing review and editing. JBi: writing - initial draft and review and editing. All authors contributed to the article and approved the submitted version.

## Conflict of Interest

The authors declare that the research was conducted in the absence of any commercial or financial relationships that could be construed as a potential conflict of interest.
